# Personal

**Published:** 1890-08

**Authors:** James Truman


### Editorial.

### PERSONAL.

The change in the management of this journal was fully announced in the last (July) number. There remains, therefore, but a word from myself on assuming the editorial control. The exigencies of the situation seemed to force this work into my hands, and, with reluctance, I consented to bear burdens not wholly unfamiliar.

It is not necessary that any policy should be outlined. The course of this journal will remain as it has in the past, an earnest factor for the elevation of the profession. It will be outspoken when necessary, and will hold, it is hoped, a positive but impersonal tone on all controverted questions.

With a full knowledge of the fallible character of all human efforts, the work is entered upon with a consciousness of possible mistakes, errors of omission and commission; but this feeling is accompanied by the assurance of the co-operation of the best element in the dental profession. Without this the work of this journal would be valueless; indeed, would have no excuse for an existence.

While it is not expected that any radical changes will be made in the arrangement of the matter, it is anticipated that improvements will be introduced as time and experience indicate their value or necessity.

The work of such a journal needs and must receive the earnest thought of the best writers and investigators of this specialty. For them it was instituted, and, as their organ, it should be the receptacle of the work of their brains and hands. It should publish the proceedings of every organized society in the United States, and, it is hoped, it will continue to receive a fair proportion of the work of the associations abroad.

With this expectation, based on the earnest support given in the past, and recognizing that all shades of thought represent that frictional activity so necessary to true progress in all directions and among all peoples, I enter upon this labor with the assured hope that the journal will remain permanently to exercise a healthy, vigorous, and lasting impression on the profession of dentistry.

JAMES TRUMAN.

